# Electrospun Poly(butylene-adipate-co-terephthalate)/Nano-hyDroxyapatite/Graphene Nanoribbon Scaffolds Improved the In Vivo Osteogenesis of the Neoformed Bone

**DOI:** 10.3390/jfb12010011

**Published:** 2021-02-05

**Authors:** Luana Marotta Reis Vasconcellos, Gabriela F. Santana-Melo, Edmundo Silva, Vanessa Fernandes Pereira, Juliani Caroline Ribeiro Araújo, André Diniz Rosa Silva, André S. A. Furtado, Conceição de Maria Vaz Elias, Bartolomeu Cruz Viana, Fernanda Roberta Marciano, Anderson Oliveira Lobo

**Affiliations:** 1 Department of Bioscience and Oral Diagnosis, Institute of Science and Technology, Sao Paulo State University, Sao Paulo 12450-000, Brazil; gabrieladsantana@yahoo.com.br (G.F.S.-M.); edsilva.am@gmail.com (E.S.); vanessafpereira@hotmail.com (V.F.P.); julianicraraujo@hotmail.com (J.C.R.A.); 2 Air Force Academy, Brazilian Air Force, Pirassununga 13630-000, Brazil; adinizrs@gmail.com; 3 LIMAV—Interdisciplinary Laboratory for Advanced Materials, UFPI-Federal University of Piaui, Teresina 64049-550, Brazil; salesandre7@gmail.com; 4 Instituto Científico e Tecnológico, Universidade Brasil, Sao Paulo 12450-000, Brazil; conceicaovazenf@hotmail.com; 5 Department of Physics, Federal University of Piaui, Teresina 64049-550, Brazil; bartolomeu@ufpi.edu.br (B.C.V.); marciano@ufpi.edu.br (F.R.M.)

**Keywords:** electrospinning, nano-hydroxyapatite, graphene nanoribbons, PBAT, bone regeneration

## Abstract

Electrospun ultrathin fibrous scaffold filed with synthetic nanohydroxyapatite (nHAp) and graphene nanoribbons (GNR) has bioactive and osteoconductive properties and is a plausible strategy to improve bone regeneration. Poly(butylene-adipate-co-terephthalate) (PBAT) has been studied as fibrous scaffolds due to its low crystallinity, faster biodegradability, and good mechanical properties; however, its potential for in vivo applications remains underexplored. We proposed the application of electrospun PBAT with high contents of incorporated nHAp and nHAp/GNR nanoparticles as bone grafts. Ultrathin PBAT, PBAT/nHAp, and PBAT/nHAp/GNR fibers were produced using an electrospinning apparatus. The produced fibers were characterized morphologically and structurally using scanning electron (SEM) and high-resolution transmission electron (TEM) microscopies, respectively. Mechanical properties were analyzed using a texturometer. All scaffolds were implanted into critical tibia defects in rats and analyzed after two weeks using radiography, microcomputed tomography, histological, histomorphometric, and biomechanical analyses. The results showed through SEM and high-resolution TEM characterized the average diameters of the fibers (ranged from 0.208 µm ± 0.035 to 0.388 µm ± 0.087) and nHAp (crystallite around 0.28, 0.34, and 0.69 nm) and nHAp/GNR (200–300 nm) nanoparticles distribution into PBAT matrices. Ultrathin fibers were obtained, and the incorporated nHAp and nHAp/GNR nanoparticles were well distributed into PBAT matrices. The addition of nHAp and nHAp/GNR nanoparticles improved the elastic modulus of the ultrathin fibers compared to neat PBAT. High loads of nHAp/GNR (PBATnH5G group) improved the in vivo lamellar bone formation promoting greater radiographic density, trabecular number and stiffness in the defect area 2 weeks after implantation than control and PBAT groups.

## 1. Introduction

Electrospinning has been extensively used to produce scaffolds for bone tissue engineering (BTE) due to its ability to produce superhydrophilic, mesoporous, bioactive, and ultrathin fibers. This emerging technology can improve the absorption of integrins from extracellular matrix, vascularization, and osteogenesis [1,2,3,4].

Poly (butylene adipate-co-terephthalate) (PBAT) is an interesting biodegradable aliphatic-aromatic copolyester to produce scaffolds using electrospinning for BTE application [5,6,7,8,9,10,11,12,13], due to its degradability after a few weeks [14]. PBAT has lower mechanical and osteoindutive and osteocondutive properties and inorganic nanoparticles have been incorporated to improve these properties [14]. The main inorganic component of bone tissues is nHAp, which demonstrates excellent biological properties for promoting cell adhesion and proliferation [15]. At the same time, nHAp has been combined with different forms of nanocarbon to optimize the mechanical properties of nHAp without impairing the bioactive function of the material [16,17]. A combination of multi-walled carbon nanotubes (MWCNTs) and graphene oxide (GO) with HAp in its various forms (as nano) are attractive due to their excellent mechanical and physical-chemical properties (low mass, high surface area, and high electrical and thermal conductivity) [17,18,19,20,21,22,23]. MWCNTs can be exfoliated and exposed to GO leaves for biological applications [19]. To improve the biocompatibility of carbon nanotubes, they were exfoliated and functionalized with hydrophilic groups, forming unpacked nanotubes with a structural atomic organization similar to graphene oxide (GO) at their ends, called graphene nanofibers (GNR) [19].

Different strategies evolving synthesis of nHAp, GNR, and nHAp/GNR nanoparticles and their ability to improve biological in vitro and in vivo properties when incorporated in different amounts (from 0.1 to 5%) into different polyesters have been reported by our group [8,11,12,13,24]. However, an in vivo analysis of bone neoformation obtained by means of several methods such as bone remodeling analysis, microtomography computadorized, radiographical analysis, and biomechanical properties from bone neoformed into bone defects filled with electrospun PBAT containing high loads of nHAp and GNR/nHAp incorporated nanoparticles had not been reported yet. Recently, our group demonstrated that conductive PBAT/nHAp (containing polypyrrole) scaffolds were non-genotoxic when implanted in vivo [8] but no evidence from bone neoformation was evaluated.

Contributing to this, here we produced ultrathin PBAT fibers scaffolds tuned with high loads of nHAp (5 wt.%) and nHAp/GNR (5 wt.%) and then used them as bone grafts for tibia defects. We systematically analyzed the bone fragments after two and four weeks of implantation using histology and micro-CT. Details, correlation, and influence of different amounts of nHAp and nHAp/GNR compared to control are discussed and compared. High loads of nHAp (5%) and nHAp/GNR (5%) improved the bone neoformation.

## 2. Materials and Methods

### 2.1. Preparation of Solutions

First, nanoparticles were prepared containing different amounts of nHAp and GNR. Our group has expertise in obtaining non-cytotoxic, bactericide, and in vitro and in vivo osteogenic GNR, using a simple acid and oxygen plasma exfoliation of MWCNTs [25,26,27]. Biocompatible and with osteogenic properties nHAp/GNR nanoparticles were also developed by our group using a simple wet chemical ultrasound assisted method [28,29]. Table 1 shows details of prepared solutions. Briefly, the PBAT (Ecoflex^®^ F Blend C1200, BASF, Munich, Germany) was used at a concentration of 20 wt.% and dissolved in chloroform under magnetic stirring (Color Squid IKAMAG^®^, Koenigswinter, Germany) for 150 min. The different nanoparticles types and concentrations (Table 1) were then sonicated dispersed (5 min, ultrasound probe, Sonics VCX 500, Oklahoma City, OK, USA) in N, N-dimethylformamide (DMF, Sigma–Aldrich, St. Louis, MO, USA). All solutions were prepared under controlled humidity and temperature conditions. The solutions were mixed and magnetically stirred prior to the electrospinning process.

### 2.2. Production of Scaffolds

The entire electrospinning process was carried out with ambient temperature (21 ± 2 °C) and controlled humidity (45 ± 5%). For this, a 5 mL solution of each group was loaded into a glass syringe (BD Yale™, Burlington, MA, USA) with a needle (Inbras^®^, 23G, Inowrocław, Poland). The electrospinning process was carried out using the following parameters: voltage: 17 kV (Bertan 230, Champaign, IL, USA); distance: 10 cm; rate: 1.5 mL h^−1^ (Kd Scientific KDS-100, Swedesboro, NJ, USA) time: 60 min.

### 2.3. Characterization

#### 2.3.1. Morphological and Structural Analyses

All the produced scaffolds were characterized morphologically using scanning electron microscopy (SEM; Zeiss EVO MA10, Jena, Germany). Ultrathin fibers were collected and coated with a thin layer of gold using a sputter-coating system before analysis. The average fiber diameters were measured from the SEM micrographs (n = 100 fibers) using ImageJ^®^ software [30]. To evaluate the nHAp and nHAp/GNR nanoparticles incorporated into the PBAT matrix, the ultrathin fibers were electrospun directly onto copper TEM grids (300 mesh), for 5 s of exposition and analyzed using transmission electron microscopy (TEM; Philips CM120 TEM operating at 120 kV, Amsterdam, The Netherlands). Others characterization and more details about used nanoparticles referred to all studied groups can be seen elsewhere [11,12].

#### 2.3.2. Mechanical Properties

The elastic modulus, tensile strength, and fracture strain of the nanofibers of neat PBAT that presented the best results in vivo were measured and compared using a texture analyzer (TA. XT plus, Stable Micro Systems Ltd., Vienna, UK). Rectangular sample of the polymeric scaffolds was specifically cut to the dimensions 10.00 mm × 30.00 mm, and the thickness was measured with a micrometer with a precision of 10 μm. The samples were fixed with the probe provided by instrumentation attached to a 5 kgf loadcell. Measures were carried out at 25 °C and a strain rate of 1 mm/min (N = 3). Young’s modulus was calculated by stress-strain ratio of linear portion in graph (strain between 0 and 4%). The statistical tests were done using One-way ANOVA followed by post-test multiple Tukey comparisons. The *p* < 0.05 was considered statistically significant.

### 2.4. In Vivo Analysis

#### 2.4.1. Surgery Procedures

Male rats (Rattus norvegicus albinus; Wistar), at 3-month-old, and weighing between 400 to 450 g) were used. The experimental procedures were performed at Sao Paulo State University (UNESP, Sao Jose dos Campos, SP, Brazil) this study was approved by the Ethics in Research Committee (number: 10/2015-CEUA-ICT-CSJC-UNESP). All rats received water and food ad libitum, and were distributed for three animals per cage. The scaffolds were disinfected in 70% ethanol and then sterilized with a UV lamp for 30 min. The animal model design of this study was a randomized, prospective, controlled, and followed the recommendations of the Animal Research: Reporting In Vivo Experiments guidelines for the execution and submission of studies on animals [31]. The surgery procedures were performed as previously described in Vasconcellos et al. (2004) [32,33]. The bone defects were performed on both tibias of each animal, and mini-rolls scaffolds with 3.5 mm diameter were inserted into the defects.

The animals (n = 5) were randomly divided in experimental groups, in accordance with material used into the critical defect: control-clot, PBAT, PBATnHA5, and PBATnHA5G. The number of animals was based on previously published papers [32,33]. The soft tissues were carefully positioned and sutured using 4-0 silk thread (Ethicon/Johnson & Johnson, Sao Jose dos Campos, Brazil) and swabbed with iodinated alcohol again. The rats were inspected day-to-day for any clinical sign of possible complications or adverse reactions. The euthanize was carried out after two weeks using an anesthetic overdose administered intramuscularly and the bone fragments were submitted to radiography, microcomputed tomography histological, histomorphometric, and biomechanical analyses.

The bone fragments used for radiography and biomechanical analysis were kept in Ringer’s solution, refrigerated at −20 °C until test, while for other tests, the bone samples were fixed in 10% formaldehyde until analysis.

#### 2.4.2. Radiography Analysis

The radiography analysis was performed using the parallelism technique with the parameters of 70 kV, 8 mA, and 0.4 s of exposure time in the conventional dental X-ray machine, brand DabiAtlante, model Spectro 70XSeletronic (Dabi Atlante, Ribeirão Preto, Brazil) as described previously in [34]. The films used were Ekataspeed Plus (EP-21p-Eastman Kodak Company, Manaus, Brazil), and after radiographic process, the radiographs were scanned and assessed using the Adobe Photoshop program. The program measured the densitometry by means of the gray scale values, and increased mean values of the gray scale pixels indicate the higher maturation of the bone tissue.

#### 2.4.3. Microcomputed Tomography

Prior to this analysis the bone fragments were removed from formaldehyde, washed in current water, and immerses in 70% alcohol solution. Images from bone specimens were taken using a Micro-CT scanning (SkyScan, Kontich, Belgium, 50 kV, 800 µA). The images were collected in 360° rotation for further reconstruction using Recon v1.6.4.8 software (SkyScan). Then, the three-dimensional projections were rearranged using Data Viewer 1.4.4.0 software (SkyScan) and reassembled using CTvox 2.3 software (SkyScan). The volume of interest (VOI) was calculated from bone volume (BV), and trabecular number (Tb.N) using standard methods at 2 weeks as described previously in [13].

#### 2.4.4. Analysis of Bone Remodeling

After evaluating bone repair using microtomography, these bone fragments were submitted to histological and histomorphometric analysis. The bone fragments were dehydrated in a graded alcohol series and embedded in methyl methacrylate. Sections of critical defect area were obtained using a diamond saw in a cutting machine for hard tissues (Labcut 1010, Extec, Enfield, CT, USA) as previously described by Vasconcellos et al. (2008) [35]. During the recovery period, fluorochromatic markers of alizarin and calcein were used to observe the bone remodeling in the critical defect area. Subcutaneous injection (0.5 mL) of calcein-green (Calceína Dinâmica P.A. 10 mg/Kg, (Dinamica Quimica Contemporanea Ltda, Campinas, Brazil)) and alizarin (Alizarina (P.A. Synth), LabSynth, Diadema, Brazil) were applied to verify bone apposition.

Finally, the half of histological sections were stained with blue toluidine for histological analyses and other half was submitted to analysis in with AxioPhot microscope (Carl Zeiss, Oberkochen, Germany) using light polarized, in order to evaluated the fluorescence markers in new bone formation.

For histomorphometric analysis, the microscopic images were acquired with a Sony digital camera (DSC-S85, Cyber-shot, Sony, Manaus, Brazil) associated with AxioPhot microscope (Carl Zeiss). ImageJ^®^ software was used to quantify the amount of new bone present in defect surgery area, to compare the performance of different scaffolds and control group. ImageJ^®^ software was also used to quantify the amount of bone apposition per day.

#### 2.4.5. Analysis of Biomechanical Properties

The test was conducted in a universal test machine Emic^®^-model DL 200 MF (São José dos Pinhais, PR, Brazil), which provided a force of 50 kgf with a constant application speed of 5.0 mm/min until specimen failure. The tibias for this test were removed from Ringer’s solution and submitted to the three-point flexural strength test, according to Silva et al., 2017 [13], to verify the tissue characteristics (neoformed bone). The load was applied transversely to the long axis of the femur on its posterior face at a midpoint between the two supports, in which each specimen was placed centrally along its length on a support containing two supports (15 mm apart), with its front face down. The load carrier and the supports used are cylindrical in shape with 3-mm diameter. The statistical analysis was performed first, all data (n = 5) were submitted to one-way analysis of variance (ANOVA) followed by a Tukey’s test, which was used for multiple comparisons (GraphPad Prism software, v. 6.01, GraphPad Prism, San Diego, CA, USA). The *p* < 0.05 was considered statistically significant.

## 3. Results and Discussion

### 3.1. Characterization of Designed Scaffolds

**Figure 1:**
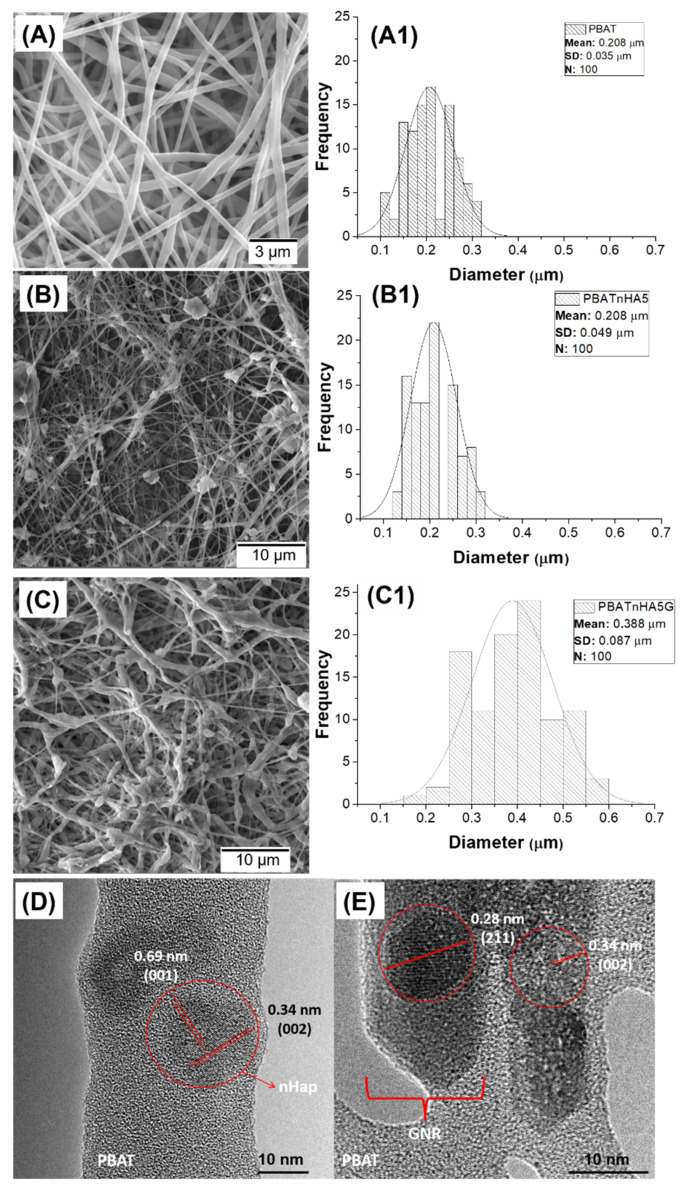
SEM image showing the ultrathin morphology of (**A**) PBAT, (**B**) PBATnHA5 and (**C**) PBATnHA5G groups. The fibers diameters analysis of (**A1**) PBAT, (**B1**) PBATnHA5 and (**C1**) Diameter distribution of PBnHA5G groups (n = 100). High resolution TEM identifying a nanometric and well-organized crystalline structures of (**D**) nHAp and (**E**) nHAp/GNR incorporated into PBAT ultrathin fibers.

Figure 1 illustrates the morphological (Figure 1A–C) diameters (Figure 1A1–C1) and structural analysis (Figure 1D,E) of all produced scaffolds, containing or not high loads of nHAp and nHAp/GNR. Figure 1A shows electrospun PBAT scaffolds, with macroporosity, interconnectivity, and ultrathin diameter (Figure 1A1, 0.208 µm ± 0.035 µm). Figure 1B presents nHAp agglomerates (PBATnHA5), macroporosity (related to space between electrospun fibers), and ultrathin fibers (Figure 1B1, 0.208 µm ± 0.049 µm) similar to neat PBAT. The macroporosity and ultrathin morphology are similar when nHAp/GNR were added (Figure 1C, PBATnH5G); however, with thinner fiber diameters (Figure 1C1, 0.388 µm ± 0.087), as expected. The TEM image of PBAT with nHAp (Figure 1D) and nHAp/GNR (Figure 1E) depicts lattice fringes and a well-defined and crystalline structure for both included nanoparticles. A d-spacings of 0.28, 0.34, and 0.69 nm can be assigned to the (211), (002), and (001) lattice planes of HAp (JCPDS # 86–1203), respectively [36,37]. Figure 1C1 charts an increase in the average diameter of the fibers, which can be attributed to the decrease in used solvent system (dielectric constant), electrical conductivity and the consequent decrease in the density of pure charge in the jet due to the presence of GNR. This variation in density and consequent change in its diameter also modifies its diffuse aspect [38,39]. The absence of agglomerated regions suggest that homogeneous distributions were achieved in Figure 1D,E in the TEM images, which clearly illustrated that the embedded GNR was tangled. In Figure 1B,C, there is no ideal alignment of its segments in the crystalline networks, because nHAp load decreased the degree of crystallinity, which is attributed to the loss of mobility of the polymer chains [12].

**Figure 2:**
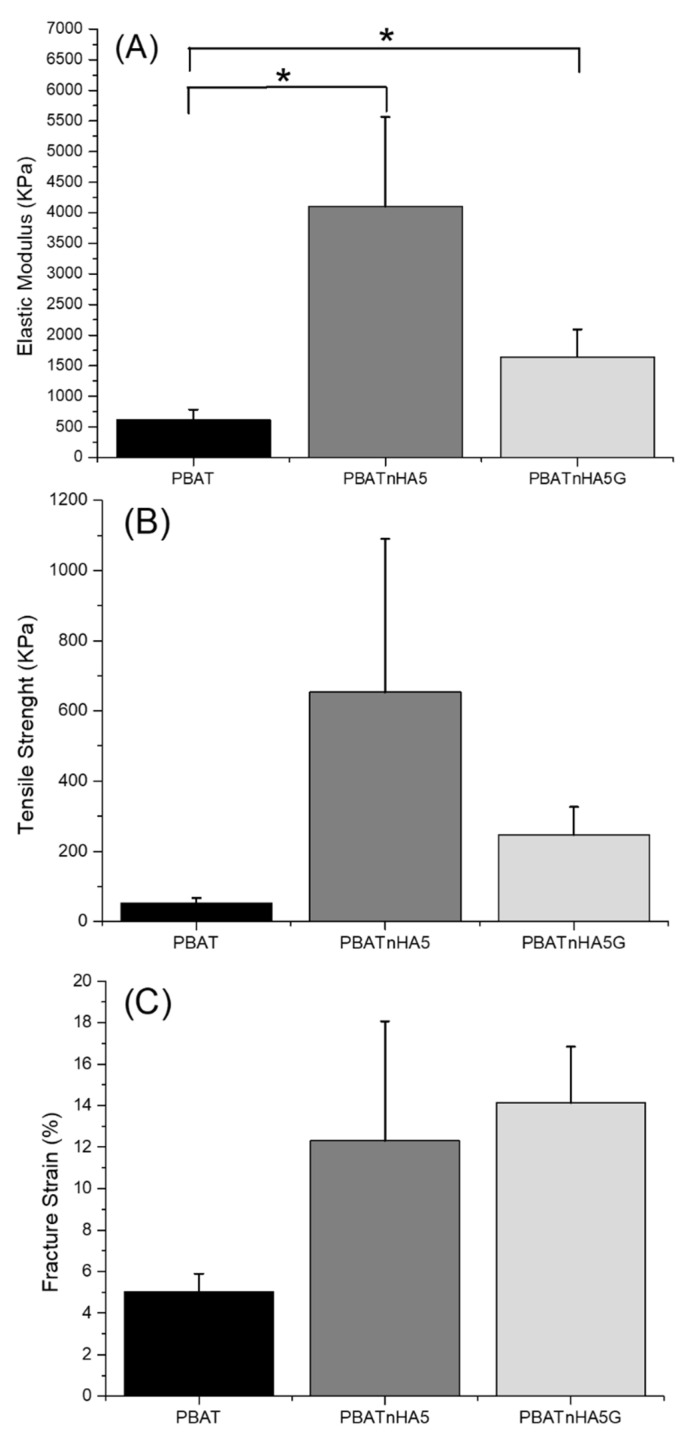
Variation in (**A**) elastic modulus, (**B**) tensile strength, and (**C**) fracture strain of designed scaffolds. The results are presented as mean and standard deviation. N = 3. * *p* < 0.05 was considered as significance.

Figure 2 compares the mechanical properties of the designed scaffolds. The mechanical properties of the scaffolds were strongly affected by the addition of nHAp and nHAp/GNR as a filler. An increase clearly occurred in the evaluated mechanical properties when high loads of nHAp (PBATnHA5 group) and nHAp/GNR (PBATnHA5G group) nanoparticles were added compared to neat PBAT ultrathin fibers. However, only elastic modulus presented statistical difference from neat PBAT (Figure 2A). No statistical difference of designed scaffolds compared to neat PBAT were observed for tensile straight (Figure 2B) and fracture strain (Figure 2C). Carbon nanotuboes and nHAp can positively or negatively influence the mechanical behavior of ultrathin fibers [40]. We identified the same concept here; however, only elastic modulus was improved for the designed groups compared to PBAT, suggesting PBATnHA5 and PBATnHA5G for bone tissue engineering applications. The improved elastic modulus of PBAT can be attributed to the favorable interactions between the polymer matrix and the nanomaterial particles as also observed elsewhere [10,11,41]. Meanwhile, the difference between PBATnHA5 and PBATnHA5G can be addressed by the addition of GNR, which may be responsible for the presence of stress concentration centers, decreasing their resistance.

We also carry out specific mechanical tests to prove that the one biomaterial can be used as a bone substitute, since its mechanical resistance must be compatible with that of the bone [42,43]. The measurements of fracture strain demonstrated that for the group that contained GNR, there was an increase due to the stiffness of the electrophilized fibers, evidencing the reinforcement of the effect of its composition, because they restrict the segmental movements of neighboring polymer chains [11,44].

### 3.2. Bone Repair Analysis

Many bone substitutes were tested and exhibited a lower percentage of bone neoformation than autogenous bone graft [45]. However, this autogenous graft procedure has disadvantages and presents important limitations, such as the risk of rejection or disease transmission [46]. Thus, an alternative bone graft made of synthetic materials is need to replacement it [47]. The advantage of synthetic biomaterials in bone tissue regeneration as an alternative to bone grafts is considerable, as they do not damage healthy tissues, do not increase the risk of contamination, and are commercially available [48]. Three-dimensional scaffolds have gained interest in bone regeneration because they can act as structures to accommodate cells and support tissue growth [49], to potentially provide support for cell adhesion, proliferation, and migration [50]. From this, polymeric scaffolds with adequate strength, rate of degradation, porosity, microstructure, shape, and size have been synthesized [10,51,52].

Polymers have been used as biomaterial to induce bone neoformation in bone defect areas. In this study, nanofibers based on PBAT, associated with nHAp and GNR, were developed and produced. They exhibited fine fibers with well-defined arrangements and allowed bone neoformation. Although few studies have focused on the use of PBAT and its nanocomposites in health [10,53], PBAT has great potential for industrial and environmental applications as well as for possible uses in tissue engineering [52,54]. In this study, the group that received PBAT or PBATnH5 as fill material achieved similar results than the control group (*p* > 0.05), while the PBATnH5G composite showed better results than control group in radiographic density, trabecular number and stiffness test.

**Figure 3:**
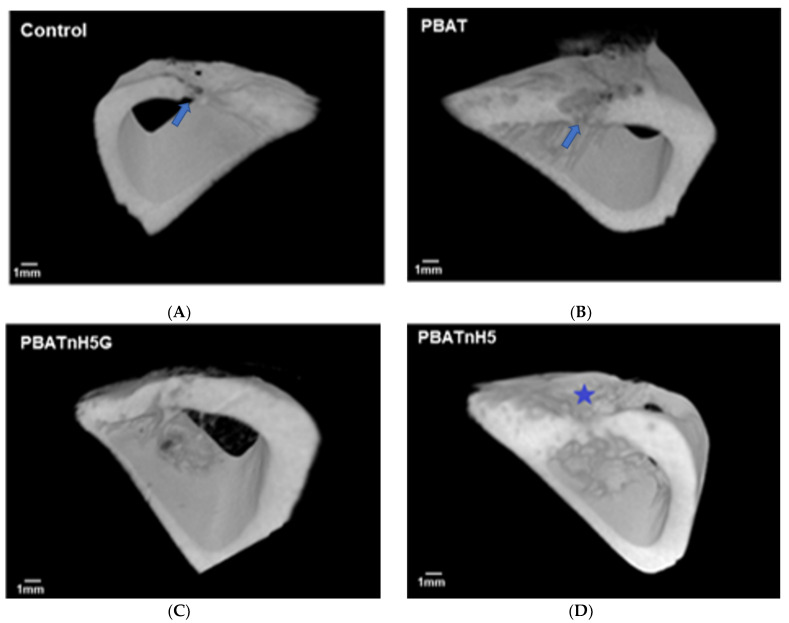
Representative 3D micro-CT images (microcomputed tomographic images) of the tibia areas defects after 2 weeks of scaffolds implantation. (**A**) Control (**B**) PBAT (**C**) PBATnH5G (**D**) PBATnH5. One depression of bone was observed in most of the other groups (☆) as illustrated in figure D. Control and PBAT group no filled the total area of thickness of tibia (→). In the area of the bone defect, bone repair can be observed by the presence of compact trabeculae that put together the defect margins on PBATnH5G group.

The micro-architecture bone observed by micro-CT and mechanical strength of bone regeneration was influenced by the biomaterials used for filling the bone defect, with an association of nHAp and GNR. In the 3D images obtained (Figure 3) by microtomography, all groups presented bone repair in the defect area, but the PBATnH5G group exhibited better bone regeneration with reconstruction of tibia thickness, as observed in Figure 3C. In the control and PBAT groups, the bone tissue was not filled in the total area of defect, as incomplete bridge bone in tibia can be observe (Figure 3A), while in PBATnH5 group completed bridge bone was observed, but with lower thickness than preexistent bone. Finally, the PBATnH5G presented completed bridge bone, and similar thickness as preexistent bone.

**Figure 4:**
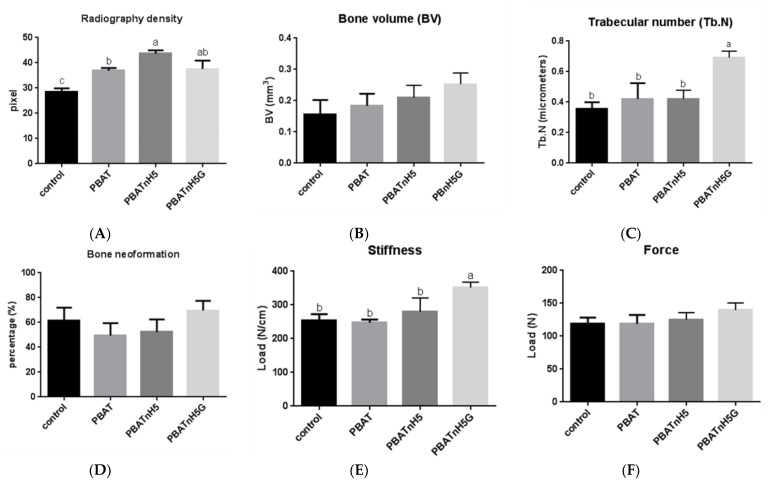
Graphics of in vivo analyses and flexural testes of new bone formation into tibia rat critical defect model: (**A**) radiography density. (**B**) Bone volume parameter microtomography analyses (BV). (**C**) Trabecular number parameter microtomography analyses (Tb.N). (**D**) Histomorphometric analyse: bone neoformation (%). (**E**) Stiffness parameter biomechanical test. (**F**) Force parameter biomechanical test. Statistical differences are showed using different letters for *p* < 0.05 (ANOVA, post-test Tukey).

Scaffolds incorporated with nHA and GNR exhibited the best results in this study although without complete statistical difference. Recently our group showed that PBAT/nHA scaffolds increased bone repair [12]. In this study, the PBATnH5 group improved bone repair, but without statistical difference (*p* > 0.05) from the control and PBAT groups when microtomography and histomorphometry were evaluated. However, the radiography density value was higher in this group than control and PBAT groups (Figure 4A), and statistical difference was observed (*p* < 0.05).

The incorporation of carbon nanotubes into the polymer matrix also promotes cell attachment and proliferation as well as impacts cell differentiation and bone regeneration [55]. Recently its positive influence was reported in a review about this material [55]. In the present study, the microstructural parameters of bone volume (Figure 4B) and trabecular number (Figure 4C) of micro-CT in bone repair were evaluated, and the PBATnH5G promoted better bone repair, while that the control group had the lowest value; nevertheless, the statistical difference occurred only in the trabecular number parameter (*p* < 0.05) between this group and the other groups. The best bone neoformation in PBATnH5G filled defect was also observed by radiographic density (Figure 4A) and histomorphometry (Figure 4D) but statistical difference with control and PBAT group (*p* < 0.05) it was observed only about radiographic density. Yuchao et al. (2019) reported better results of bone formation in graphene oxide PLA scaffolds than nHa PLA and control PLA groups, but with statistical difference (*p* < 0.05) [56].

The bone repair obtained with PBATnH5G filled also demonstrated the highest values for mechanical characteristics, but without statistical difference among the groups when the force parameter (Figure 4E) was evaluated (*p* > 0.05). However for the stiffness parameter (Figure 4F), the tissue bone formed in the area defect filled with PBATnH5G scaffold showed better results, statistically different from other groups (*p* < 0.05) as described also in Eivazzadeh-Keihan et al. (2019) [55].

**Figure 5:**
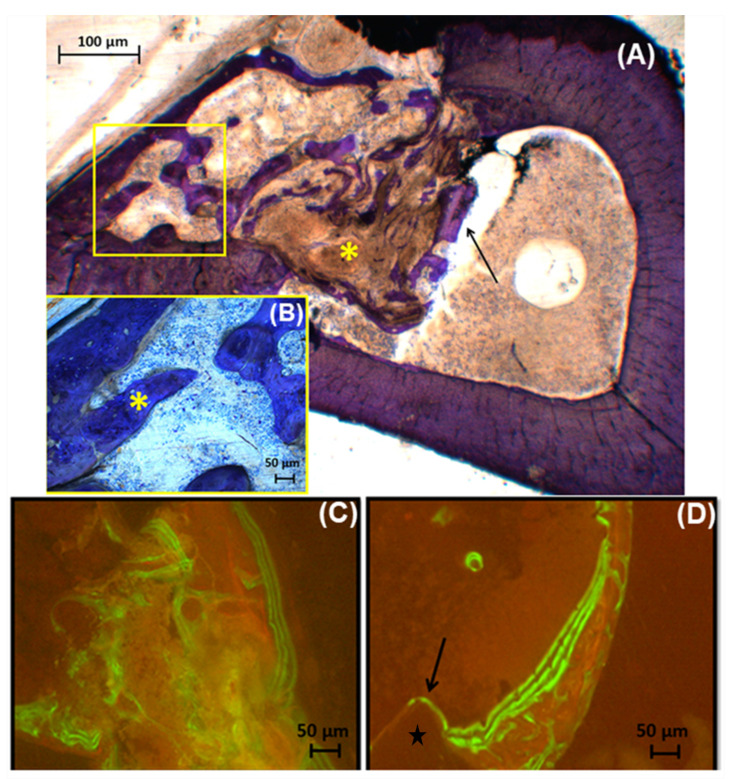
Histology bone defects of bone repair with PBnH5G: (**A**) panoramic view of the tibial defect, presence of immature trabecular bone (→), biomaterial (*) into the defect, both permeated by cells; (**B**) details of anterior figure, new bone trabeculae (*); (**C**) Fluorescent line into critical defect area; (**D**) It is observed that the preexistent bone does not fluorescent (☆), a sign that the bone neoformation was concentrated in the surgical wound, there is evident remodeling line between new bone formation and preexistent bone (→).

Figure 5 contains the histologic (Figure 5A,B) and fluorescent (Figure 5C,D) images, which are representative of groups since there was not difference statistical among them in histomorphometric analyses (Figure 4D). The histological analyses observed immature bone trabeculae permeated with cells that mix with the region of the bone marrow in all groups, and the residual presence of the material that was not reabsorbed. Figure 5C illustrates the bone neoformation on area defect, and fluorescent lines indicate active new bone formation and bone remodeling. Figure 5D exhibits longitudinal lines that indicate the formation of the bony bridge that goes from the pre-existing bone towards the center. Thus, porous scaffolds contribute to bone neoformation, promoting greater osteogenic differentiation [57]. The results of the analysis of the scaffolds of PBnHaG group have great potential to regenerate critical bone defects due to osteoconductivity of the cells and the bioactivity of scaffolds, which is an important feature for tissue regeneration [58].

The PBATnH5G group showed highest values of bone repair, often with statistical difference when compared to the control and PBAT groups (*p* < 0.05). In all the tests the PBATnH5G group exhibited the best results.

## 4. Conclusions

In conclusion, the current study successfully demonstrated the potential of scaffolds for bone regeneration to use as a promising biomaterial. High loads of nHAp and nHAp/GNR were successfully incorporated into PBAT matrices without changing the ultrathin diameter. The addition of nHAp and nHAp/GNR improved the elastic modulus of ultrathin fibers. The attractive properties of PBATnHA5 and PBATnHA5G (mean diameter, µCt, histomorphometrically, microstructurally, and biomechanically) positively provided support for bone proliferation and migration, confirmed by micro-CT images. The PBATnH5G group also demonstrated more effective bone formation, promoting greater trabecular number and stiffness in the defect area 2 weeks after implantation.

## Figures and Tables

**Table 1:** Composition and names of the electrospun experimental groups.

Groups	Named	nHAp (%)	GNR (%)
PBAT	PBAT	-	-
PBAT/nHAp	PBATnH5	5	-
PBAT/nHAp/GNR	PBATnH5G	5	1

## Data Availability

All raw data from characterization are available from the corresponding author upon request.
